# SPiRIT study protocol (Shoulder Pain: Randomised trial of Injectable Treatments): a randomised feasibility and pilot study of autologous protein solution (APS) vs corticosteroids for treating subacromial shoulder pain

**DOI:** 10.1186/s40814-023-01425-9

**Published:** 2024-01-17

**Authors:** A. Howard, A. Woods, I. Rombach, J. Achten, D. Appelbe, A. Athwal, E. Jones, K. Draper, S. Gwilym

**Affiliations:** 1 Oxford Clinical Trials Research Unit, Oxford Trauma, Kadoorie Centre, NDORMS, University of Oxford, Oxford, England; 2 University of Oxford, Joint Research Office, 1St Floor, Boundary Brook House Churchill Drive, Headington, OX3 7GB England; 3 NIHR Leeds Biomedical Research Centre, Chapel Allerton Hospital, Leeds, UK; 4 Academic Department of Trauma & Orthopaedics, School of Medicine, University of Leeds, Leeds General Infirmary, Leeds, UK

**Keywords:** Shoulder pain, Injection, Steroid, Autologous protein solution

## Abstract

**Background:**

The management of subacromial shoulder pain represents a significant challenge and is typically managed through either physiotherapy, joint injection or surgical intervention. Recent surgical trials have questioned the efficacy and there is a need to improve the evidence base for the non-surgical management of this condition.

The study aims to provide evidence of the feasibility of conducting a randomised controlled trial to compare the efficacy of autologous protein solution (APS) against the current standard of care, corticosteroid injection (CSI) for subacromial shoulder pain. Autologous protein solution (APS) is a blood-derived biological injection which has been shown to have anti-inflammatory effects.

**Methods:**

A parallel-group two-arm randomised control trial will be conducted, comparing APS and CSI for shoulder pain. Fifty patients will be recruited. Feasibility will be assessed by examination of the conversion rate of eligible participants to the total number of participants recruited, whether it is possible to collect the appropriate outcome measures and the levels of retention/data compliance at follow-up dates.

**Discussion:**

CSI is the mainstay of conservative management of subacromial shoulder pain. Trials and systematic reviews have reported differing conclusions, but the consensus view is that any benefits seen from CSI use are most likely to be short-term and there remains a significant number of patients who go on to have surgical intervention despite CSI.

Biological injections, such as APS are being increasingly used, in the anticipation they may offer improved longer lasting outcomes for shoulder pain. However, the evidence to demonstrate the comparative efficacy of CSI versus APS does not currently exist. If feasible, a fully powered study will offer clarity to the treatment pathway of thousands of patients each year with subacromial pain.

**Trial registration:**

The study is funded by the National Institute for Health Research–Research for Patient Benefit, NIHR 201473, Trial Registration Number (ISRCTN12536844: SPiRIT. Shoulder pain: randomised trial of injectable treatments–date of Registration 15/9/2021). Protocol Version V1.0_30Jul2021. IRAS Project ID: 294,982.

## Background

Shoulder pain accounts for 1–2% of all adult consultations with a GP [1]. Of this shoulder pain, around 70% is subsequently attributed to pain arising from the tendons which move and stabilise the shoulder; the rotator cuff. Most commonly these problems are due to inflammation and degeneration of the tendons [2]. Shoulder pain does not always have a favourable outcome with current treatments. Only 59% of patients treated in primary care showing a complete recovery within 6 months [3]. Symptoms may be disabling in terms of the patient’s ability to carry out daily activities at home and the workplace, resulting in time off work. This poses a substantial burden to the individual and society [4–6]. In the USA, the annual financial burden of shoulder pain management has been estimated to be $3 billion [7].

A mechanical explanation for shoulder pain has previously been favoured, whereby contact occurs between the rotator cuff tendons and the overlying bone. This ‘rubbing’ process was felt to result in inflammation of the rotator cuff tendons and nearby structures including a fluid-filled sac called the subacromial bursa. Treatments have historically been directed at reducing this inflammation and rubbing process, either by injections of corticosteroids (to address the inflammation) or surgical intervention to remove some of the bone, which was felt to be rubbing on the tendon. Evidence for the efficacy of both surgical and non-surgical treatments of shoulder pain is limited. A recent publication by the British Elbow and Shoulder Society (BESS) and the British Orthopaedic Association (BOA) highlighted the lack of evidence for a number of interventions used to treat subacromial shoulder pain and the need for research in this area [7]. Given the large number of patients who present to general practice with subacromial shoulder pain, any developments in the treatment of this chronically painful condition will improve the care of thousands of patients each year in the UK.

Currently, Corticosteroid injections (CSI) remain the mainstay of initial treatment in the majority of cases of shoulder pain presenting to both general practice and secondary care. The efficacy of CSI has been tested in a number of trials and subsequently through systematic review. These have reported differing conclusions, but the consensus view is that any benefits seen are most likely to be short-term and there remains a significant number of patients who go on to have surgical intervention despite CSI. In addition to the lack of strong evidence towards the efficacy of CSI, there have also been theoretical and lab-based deleterious effects of corticosteroids on tendon biology reported. CSI might impair the potential for intrinsic tendon repair mechanisms but it may increase the risk of subsequent tendon tearing.

A contemporary understanding of the biology of shoulder tendons, however, gives potential targets for new pharmaceutical or biological treatments. An example of such a strategy is the use of injectable platelet-rich plasma (PRP) or Autologous Protein Solution (APS). PRP is a concentrate of platelet-rich plasma protein derived from whole blood, centrifuged to remove red blood cells. Basic science studies have consistently shown the beneficial effects of PRP on tendons including increased tendon cell proliferation, increased expression of anabolic genes and proteins, and reduced tendon inflammation [8–10]. Unfortunately, these in-vivo findings have not translated to reliable clinical application when subject to clinical trial [11]. APS is prepared using a single-use device that produces a cell concentrate from autologous blood. Conceptually, APS and PRP are very similar as they both aim to isolate anti-inflammatory cytokines and anabolic growth factors from a patient’s own blood, allowing this to be reintroduced at the site of pain. Unlike PRP systems, the APS production process preferentially concentrates anti-inflammatory cytokines production by white blood cells, including IL-1 receptor antagonist and TNF receptor inhibitor [12]. The use of APS is expanding both in the UK and worldwide. Feasibility studies investigating APS in humans, in the context of knee arthritis have been conducted. The treatment also demonstrated a favourable safety profile and was well tolerated [13]. In a further study of patients with arthritis, 46 patients were randomised to receive a single ultrasound-guided injection of APS into the knee, or a single saline injection. Patient-reported outcomes showed improvement in function and reduction in pain. The average change from baseline to 12 months in WOMAC pain score was 65% in the treatment group and 41% in the control group (*p* = 0.02) [14].

### Aims and objectives

There is a recognition that robust evidence must be produced before blood-derived therapies are further introduced into orthopaedic clinical practice [15]. These therapies are freely available in private practice and their use both in the UK and worldwide is increasing. No similar work currently exists to assess the efficacy of APS in treating shoulder pain. Further, to date, no randomised controlled trial (RCT) has investigated a comparison of CSI and APS for shoulder pain. The best means for evaluation of the clinical and cost-effectiveness of APS versus the current standard of care, CSI, would be to undertake a fully-powered multicentre RCT. However, there are uncertainties, specifically regarding recruitment rates and participant retention, that need to be assessed before undertaking a large-scale RCT.

This feasibility study trial aims to:

- Examine the conversion rate of eligible to randomised participants and total number of participants recruited.
- Investigate the levels of retention and data compliance at follow-up dates.
- Assess whether it is possible to collect the appropriate outcome measures (PROMIS Physical Function (upper extremity), PROMIS pain interference questionnaire[16], Oxford Shoulder Score (OSS) [17], Pain visual analogue score (VAS), EQ-5D-5L score [18], Work Productivity Impairment Questionnaire (WPAI) [19] and patient/hospital reported resource use including referral rates for shoulder surgery) at baseline, 3 months and 6 months.
- Gather data on any complications of either intervention at 6 months.

## Methods

### Trial design and setting

**Fig. 1:**
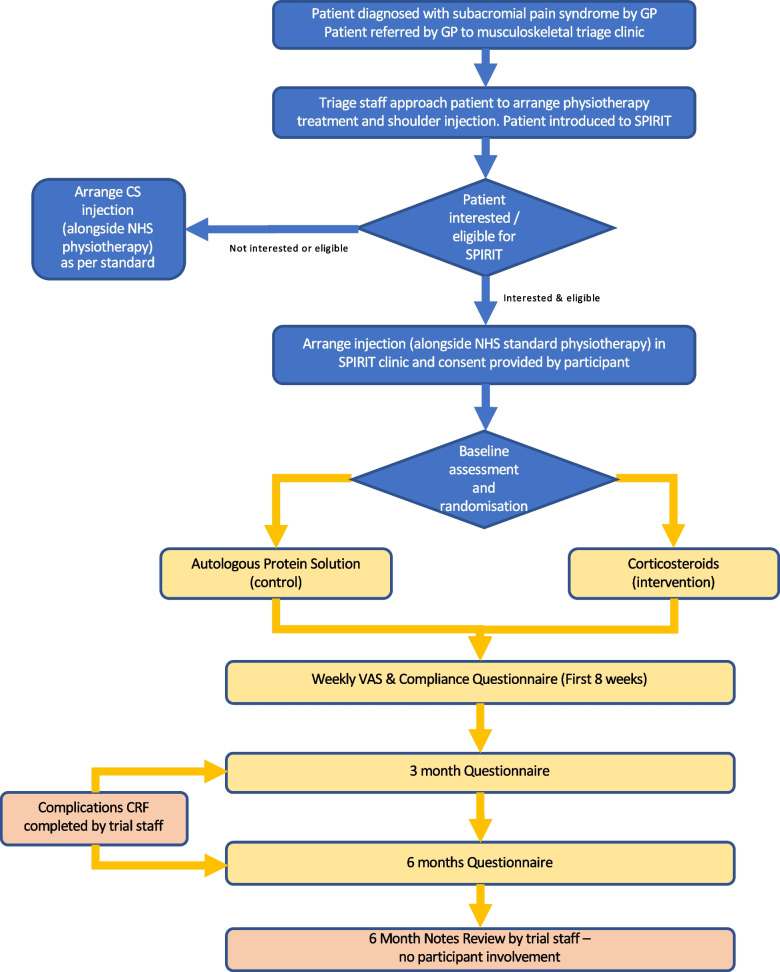
Study flowchart

This is a feasibility study of a participant-blinded, parallel group randomised controlled trial. Participants will be followed up clinically as per NHS standard of care. They will also be followed up via questionnaires by the central trial team for a period of 6 months post-treatment. We will recruit 50 participants from two NHS Musculoskeletal (MSK)–triage centres, one in Oxford and the other in Leeds from an existing standard treatment pathway (Fig. 1). The trial will be conducted in accordance with the UK Policy Framework for Health and Social Care Research, the applicable UK Statutory Instruments, (which include the Data Protection Act 2018) and the principles of Good Clinical Practice. The study is following protocol version 1.0 (30 July 2021), Oxford University, Oxford, England, is the sponsor for this trial. The study is funded by the National Institute for Health Research–Research for Patient Benefit, NIHR 201473, Trial Registration Number (please complete).

### Recruitment of participants

As part of usual care for the management of shoulder pain, MSK-triage clinicians will initially prescribe structured physiotherapy to all patients. In addition to structured physiotherapy, patients will be offered an injection into the subacromial space at a separate appointment, as per current clinical practice. GP referrals to the MSK centres were triaged and then eligibility screening then performed following the initial assessment (Fig. 1).

The trial eligibility screening will be undertaken, Fig. 1, and during patient contact to arrange a time for their appointment, they will be asked about their willingness to take part in the study. The SPIRIT team will then contact the patient separately if they are willing to participate and these individuals will be sent a trial invitation letter and participant information sheet (PIS).

### Consent process

The informed consent process will commence when the usual-care clinician confirms the patient should be treated with a therapeutic injection, the participant meets the eligibility criteria for the SPIRIT trial and is willing to take part. They will be informed that they can freely withdraw from the trial or any part at any time without giving a reason.

### Participant eligibility

#### Inclusion

- Participant is willing and able to give informed consent for participation in the study.
- Male or Female, aged 18 years or above.
- Clinician believes the patient may benefit from injection treatment.

### Exclusion

- Participants with a history of significant shoulder trauma (fracture or dislocation in last 5 years).
- Previous shoulder surgery on the affected shoulder.
- Contraindications to APS therapy or CSI.
- A pre-existing neuro-degenerative and/or vascular condition that affects the function of the shoulder.
- Received CSI/APS injection in 2 months prior to randomisation.
- The participant is unable to follow trial procedures.
- Patient does not have access to email/smartphone directly or indirectly.

### Ineligible participants

Screening logs will be kept at each site to determine the number of patients assessed for eligibility and reasons for exclusion. In addition, the number of eligible and recruited patients, and the number of patients who decline consent or withdraw will be recorded. Verbal consent will be requested to store anonymous research data, for example, reasons why they were not eligible, to help inform any future trial.

### Randomisation and blinding

Once informed consent has been obtained, eligibility confirmed and baseline data collected, participants will be randomised at the level of the individual on a 1:1 basis to either a Corticosteroid injection or an Autologous Protein Solution injection. Randomisation will be performed via a secure web-based service provided by the Oxford Clinical Trials Unit. Randomisation will be stratified by centre, duration of symptoms (≤ / > 6 months) and baseline PROMIS pain interference scores.

To avoid bias in the delivery of the intervention and completion of participants’ reported outcomes, the participants are to be kept blind about the treatment that is allocated. This blinding will be achieved by collecting the blood sample required for APS (55 ml), from both groups of patients. In the intervention group, this blood will be used for the preparation of the APS; in the control group, this blood will be sham prepared as APS, but discarded. The injections will then be performed using opaque syringes to prevent unblinding the patient.

Both the APS and the comparator group will undergo structural physiotherapy, the timing of which will begin either before or after the injection has taken place, as per local protocols. Both injections, where possible, will be undertaken under ultrasound guidance.

#### The APS intervention

After the consent and randomisation processes, a 55-ml sample of blood will be obtained and taken to the sample preparation area. The blood will be used for the preparation of the APS injection. The solution will be created as per the manufacturer’s guidelines. It is a two-step process taking 15–20 min—firstly, the blood is separated by centrifuging it, after which it is concentrated in specialised tubes. The total volume of the resultant APS is approximately 3 mls, and does not contain any local anaesthetic. This solution will be delivered in an identical manner to the control treatment but provided in a ‘blinding syringe’ (non-transparent sides).

The APS injection kit is manufactured on behalf of Zimmer Biomet, a medical device manufacturer, it has the trade name of nSTRIDE. nSTRIDE is fully licenced for use in the UK, and the processing machines have the appropriate CE markings.

### Comparator group

We are not proposing any change of standard care for the CSI group, save that to maintain participant blindness to the intervention the participants will have 55 ml sample blood taken, which will be taken to the sample preparation area. This blood will be discarded in the sample preparation area whilst a sham-centrifuge process is performed in order to maintain participant blinding. The control participants will then receive the CS injection, which contains Depo-medrone (40 mg) mixed with 3 mls of 0.5% bupivacaine local anaesthetic, administered using standard aseptic techniques, but provided in a ‘blinding syringe’ (non-transparent sides).

### Post-injection follow-up

For both treatments, immediately after the injection the participant will receive standard advice and can immediately resume normal daily activities. After 6–8 weeks if no significant medium-term benefit, as defined by usual clinical assessment, is reported, the patient will be referred to secondary care to discuss alternative treatment options as per standard care pathways.

To ensure that the participant’s clinical team know what treatment they have already received as part of the study when they revert back to their standard care, the participant’s medical notes will state their involvement in the SPIRIT trial and that the site research team should be contacted prior to further treatment commencing to establish which intervention the participant originally received.

### Primary outcome

The primary outcome is the feasibility and acceptability which will be assessed by the rate of patient conversion from eligibility for the study to randomisation. Numbers of screened ineligible patients will be noted to ensure recruitment for the main trial is feasible within this patient population. Numbers of screened eligible participants declining versus converted to randomised participants will be noted to ensure that the conversion rate of eligible to randomised participants is accurately estimated.

### Secondary outcomes

Levels of participant retention and data compliance will be measured by loss to follow-up, missing data and withdrawal at the end of the trial will be collected. Data will be collected on outcomes that we plan to collect in the definitive trial. While this feasibility trial is underpowered to detect meaningful differences, an attempt can be made to ensure all the relevant data are able to be collected. Further, the current favoured outcome measure for the full RCT trial, PROMIS [16], can be assessed as an effective primary outcome and used in the power calculations for the definitive trial.

The following clinical scores will be collected; Oxford Shoulder Score (OSS) [17], Pain visual analogue score (VAS), EQ-5D-5L score [18], Work Productivity Impairment Questionnaire (WPAI) [19] and patient/hospital reported resource use including referral rates for shoulder surgery at baseline, 3 months and 6 months. We will gather data on any complications of either intervention at 6 months.

### Data collection

At baseline demographic data, patient function and pain data using the PROMIS physical function, PROMIS Pain interference, Oxford Shoulder Score, VAS and Work Productivity Impairment questionnaires will be collected after the participant has provided consent. Participants will also be asked to complete the EQ-5D-5L health-related quality-of-life questionnaire to indicate their typical health status. All case report forms (CRFs) including screening, consent, randomisation and baseline assessment will be completed online on the REDCap database.

Data collection will be divided into two phases, early and late phase follow-up:

- *Early*—participants will receive a weekly text/email/phone call (according to participant preference) up to week 8 post-randomisation with a link to a visual analogue scale (VAS) asking them to indicate their level of pain in the previous 24 h and whether during that week if they have taken any painkillers for their injury.
- *Late*—at 3 and 6 months post-randomisation, participants will be contacted by the Oxford central study office via automated SMS or email from the REDCap database and invited to complete the PROMIS, OSS, EQ-5D-5L, VAS, WPAI, health resource use and complications questionnaires.

Complication data will be collected from patients at 3 and 6 months post-randomisation. Complications will be patient-reported via their 3- and 6-month questionnaires and verified by research nurses at the site. At 6 months post-randomisation the site staff will be asked to complete a medical notes review to ensure all expected complications are recorded.

### Adverse event reporting

Both APS and CSI are licenced for use in the UK for shoulder injections and will be delivered in the usual manner. We will collect patient-reported adverse events at 8 weeks, 3 months and 6 months.

### Serious adverse events

All serious adverse events (SAEs) will be reported in an expedited manner. The following has been defined as any untoward medical occurrent that results in death; is life-threatening; requires inpatient hospitalisation or prolongation of existing hospitalisation; results in persistent or significant disability/incapacity; consists of a congenital anomaly or birth defect. Other ‘important medical events’ may also be considered a serious adverse event when, based upon appropriate medical judgement, the event may jeopardise the participant and may require medical or surgical intervention to prevent one of the outcomes listed above.

Foreseeable SAEs and adverse events not defined as serious that are related to the interventions will be recorded by participants or site staff but will not need to be reported immediately. Foreseeable adverse events to be recorded as complications include: septic arthritis; dizziness; nervousness; facial flushing; insomnia; flare-up of pain intensity at the injection site; Injection site skin pigmentation; subcutaneous fat atrophy.

### Statistical considerations and data analysis

Fifty participants will be recruited for this feasibility study. This sample size will be sufficient to estimate the rate of recruitment (i.e., participants randomised out of those screened) and retention.

Fifty participants should provide a robust estimate of the standard deviation around the PROMIS upper limb physical function to be used in the sample size calculation for a definitive trial. Specifically, to prepare for a definitive trial with an anticipated small standardised difference (0.1–0.3) and 80% power, a pilot study sample size of 40 participants (20 per arm) would be sufficient to estimate reliable input parameters for the sample size estimation of the definitive trial [20]; 50 participants will allow for up to 20% loss to follow-up.

The data analysis will be mainly descriptive for the feasibility trial, focussing on the primary outcome, i.e. the conversion rate of eligible to randomised participants, and the total number of participants recruited, as well as on retention and compliance data. Data on patient-reported outcomes, adverse events and complications will also be presented.

Descriptive statistics will be presented for all patient-reported outcomes at baseline and follow-up. Complications and adverse events/serious adverse events reported during the trial follow-up will also be summarised. Data will be presented by the treatment arm and overall. Mean and standard deviation (or median and interquartile range if non-normally distributed) for continuous variables and the number and percentage of participants in each group for binary or categorical variables will be presented.

Differences in outcomes between treatment arms at the follow-up time points will be presented as mean differences with corresponding 90% confidence intervals for continuous data, and risk differences and odds ratios with 90% confidence intervals for binary data, where appropriate. If sufficient outcome data are available, differences will be adjusted for the duration of symptoms (< = / > 6 months) and baseline scores for continuous variables, where applicable, and derived from multilevel mixed-effects linear or logistic regression models. If insufficient data are available, unadjusted differences will be presented.

Summaries by treatment arm will be based on the intention to treat (ITT) population. The ITT population will include all participants with available data for at least one of the follow-up timepoints up to and including 6 months follow-up in the randomised groups to which they were allocated regardless of the treatment they received.

### Trial oversight and management

The Trial Management Group (TMG), which includes a patient member will meet regularly, at least once a month, to ensure successful delivery of the trial. The TMG will monitor the rate of recruitment, any departure from the protocol or other safety concerns.

The trial statistician and the information specialist will be closely involved in setting up data capture systems, design of databases and clinical reporting forms. As this is a low-risk, small feasibility study no Trial Steering or Data and Safety Monitoring Committee will be convened.

### Progression criteria

The progression criteria to a full trial will be assessed against the rate of recruitment, with success defined as the recruitment of 50 patients into the feasibility trial.

## Discussion

Shoulder pain is common, and its treatment represents a challenge. Shoulder pain has a significant effect on the individuals’ activities of daily living. One of the mainstays of treatment for this condition is a CSI into the shoulder, but the evidence around the medium-term efficacy of this treatment is absent. Further, the evidence around the use of APS injection is underdeveloped but potentially represents an effective treatment for a large number of patients with subacromial pain. This feasibility will guide the appropriateness or otherwise of funding a fully-powered intervention trial comparing CSI and APS.

## Data Availability

Not applicable.
